# Gelatin-Thrombin Matrix: A New and Simple Way to Manage Recurrent Epistaxis in Hematology Units

**DOI:** 10.1155/2013/851270

**Published:** 2013-05-23

**Authors:** G. Buiret, M. Pavic, J. C. Pignat, F. Pasquet

**Affiliations:** ^1^Service d'ORL, CH Valence, 179 boulevard du Maréchal Juin, 26000 Valence, France; ^2^Service d'ORL et Chirurgie Cervicofaciale, Hôpital de la Croix-Rousse, Hospices Civils de Lyon, 103 Grande rue de la Croix-Rousse, 69004 Lyon, France; ^3^Service de Médecine Interne et Oncologie, HIA Desgenettes, 108 Boulevard Pinel, 69003 Lyon, France

## Abstract

*Introduction*. In case of thrombopenia and/or thrombopathy, epistaxes are very difficult to manage. *Case Series*. Two patients, one with a thrombocytopenia, the other with a thrombopathy, were hospitalized because of repeated active epistaxes after failure of packing. Both patients were successfully treated with an application of Surgiflo without side effects and left the hospital without recurrence of epistaxis. *Discussion*. Being a subject of many studies dealing with epistaxis, Surgiflo is a simple treatment that seems to be very effective and without side effects to treat acute epistaxis in fragile patients with coagulation disorders. Prospective studies of tolerance and efficiency in such situations should be performed.

## 1. Introduction

Epistaxes are frequent in adults [1]. Local (e.g., trauma, cancer, etc.), general (e.g., hypertension, anticoagulants; etc.), and idiopathic causes are distinguished [2], and these causes must always be managed. Stopping the blood flow is sequential, applying direct pressure to the nose, followed by anterior packing, then anterior and posterior packing, and finally performing surgical cautery under general anesthesia [3] or endovascular treatment [4]. Surgiflo, a gelatin-thrombin matrix, is being evaluated after endoscopic sinus surgery as an alternative to the painful traditional packing that is painful and traumatic for the nasal mucosa [5].

In hematology, because of thrombocytopenia and/or thrombopathy, epistaxis is more frequent and more difficult to treat due to recurrence.

Two patients, one with thrombocytopenia, the other with thrombopathy, were hospitalized for recurrent epistaxis after repeated local treatments failed. Both patients were successfully treated with the application of Surgiflo. 

## 2. Cases Presentation

Surgiflo is a dehydrated gelatin-thrombin matrix in a syringe. For endonasale use, its gelatinous consistency must be maintained to remain in the nasal cavity (reconstitution with up to 3.5 to 4 mL of sterile water). A 16 cm-long applicator is put through the nasal vestibule to the nasopharynx. Patient must make a “ke-ke-ke” sound continuously so that the soft palate joins the posterior pharyngeal wall and excludes the nasopharynx from the oropharynx. The rhinopharynx and the nasal cavity are filled in with Surgiflo from back to front.

### 2.1. Patient No. 1

Mr. C, 58 years old, was hospitalized for a bilateral anterior and posterior epistaxis. He was treated with 5-azacytidin in the Hematology Unit of HIA Desgenettes for type 2 myelodysplastic syndrome with refractory anemia and excess blasts. Even with this treatment, a pancytopenia with a deep thrombocytopenia remained. Because of an allo-immunisation (anti-HLA antibody), platelet transfusions were ineffective. Epistaxis treatment began on September 9, 2011. He underwent many effective anterior packings but epistaxis immediately recurred after each packing removal. The platelets blood count was 8000/mm^3^, and platelets transfusions could neither stop the bleeding nor increase the platelets blood count.

The patient was transferred to ENT consultation in Croix-Rousse Hospital on September 20, 2011. After packing removal, Surgiflo was applied inside both nasal cavities without local anesthesia, and the patient was transferred back to the Hematology Unit in HIA Desgenettes. He was able to leave the hospital after two days of monitoring without recurrence of bleeding. No cause of epistaxis was found during the one-month ENT consultation (particularly after nasal fiberscopy). No epistaxis recurred until the patient died from a septic shock due to neutropenia in February 2012, that is, ten months without any epistaxis.

### 2.2. Patient No. 2

Mr. H, 89 years old, was hospitalized on February 28, 2012, because of a left anterior epistaxis. He spontaneously stopped his clopidogrel treatment (aortic-bifemoral bypass 15 years earlier) twelve days earlier. He was treated by repeated left anterior packings that were effective but bleeding recurrences were immediate after each packing removal. A bilateral sphenopalatine artery cautery was performed under general anesthesia on February 29. After postoperative packing removal, the patient bled. He was transferred to the Hematology Unit of HIA Desgenettes to manage his thrombocytopenia, which was supposed to be the main cause of his epistaxis (platelet blood count of 120 000/mm^3^) and a normocytary regenerative anemia (haemoglobin 98 g/L, MCV 94.3 fL, reticulocytes at 150 000/mm^3^) with erythromyelemia and dacryocytes evoking a primitive myelofibrosis. PFA 100 was longer, proving a thrombopathy, frequently linked with this kind of myeloproliferative disorder that could explain epistaxis recurrence. The patient was transferred to the ENT unit of Croix-Rousse Hospital on March 14. After anterior packing removal, his left nasal cavity was filled with Surgiflo without local anesthesia. The patient was able to leave the hospital after two days of monitoring without bleeding. No local cause was found when the patient was seen in ENT consultation on March 27, without bleeding recurrence. The patient only complained about a nasal obstruction 15 days after Surgiflo was applied. No epistaxis recurred until November 2012, that is, eight months without any epistaxis.

## 3. Discussion

Anterior or posterior epistaxis is frequent, particularly in patients treated in oncohematology (thrombocytopenia, thrombopathy, hepatic failure, etc.). ENT surgeons (to physically stop the bleeding) and hematologists (to increase coagulation) must collaborate to manage these situations. 

Anterior packing allows stopping the majority of bleedings. The disadvantages are local infections and septic complications and, during packing removal, pain, mucosal traumatism, and bleeding recurrence. An alternative can be anterior absorbable packing such as surgicel or gelfoam. However, the placement is painful in outpatients not under general anaesthesia and is less effective for posterior bleeding. 

Surgiflo is mainly used in neurosurgery, in thoracic and vascular surgery. The reconstitution of surgiflo(r) is easy and porcine gelatin matrix allows thrombin to perform hemostasis. Surgiflo prevented bleeding after endoscopic nasal surgery in 96.7% of patients in the study of Woodworth et al. [5]. Many prospective studies are currently under way: posterior epistaxis in Spain (ClinicalTrials.gov NCT01051427), PRHéPOCE protocol in endoscopic, sinus surgery in France. The reconstitution of surgiflo(r) is easy and reconstitution is simple and needs not necessarily to be performed by an ENT surgeon (blind application without local anesthesia).

Surgiflo use avoids disadvantages of anterior packing, is effective, can be used in outpatient, and is reabsorbable. The cost of this product (119€) is offset widely by the reduction of hospitalization (1695€/d in ENT unit, 1420€/d in Hematology Unit [6]).

## 4. Conclusion

Surgiflo is a simple treatment and seems to be effective and without side effects in recurrent epistaxis, as well as being resistant to local treatment in patients with coagulation disorders (thrombocytopenia, thrombopathy, etc.). Prospective studies of tolerance and efficacy in such situations should be performed.
